# Are illusory visual phantoms seen by the motion system: Investigations utilizing the motion aftereffect

**DOI:** 10.21203/rs.3.rs-6277666/v1

**Published:** 2025-04-07

**Authors:** Loïc Daumail, Randolph Blake, Frank Tong

**Affiliations:** 1 Department of Psychology, Vanderbilt University, Nashville, Tennessee, United States; 2 Vanderbilt Vision Research Center, Vanderbilt University, Nashville, Tennessee, United States; 3 School of Psychology, Georgia Institute of Technology, Atlanta, Georgia, United States

**Keywords:** Motion perception, visual phantom illusion, perceptual filling-in, motion aftereffect

## Abstract

The constructive nature of motion perception has been highlighted in studies of the visual phantom illusion. Visual phantoms can occur when two low-contrast collinear drifting gratings are separated by a blank gap, leading to the ghostly impression of drifting stripes that extend through the gap. Although previous work has shown that phantom-inducing gratings can elicit a motion aftereffect (MAE) in the gap region, it is not known whether these MAEs arise from the perception of visual phantoms *per se*. Here, we evaluated the strength of MAEs elicited by phantom-inducing gratings and well-matched control stimuli. Either the darkest portion of the inducer gratings matched the background luminance to elicit more vivid phantoms or the mean luminance of the inducers matched the background as a control. Both phantom and control inducers predominantly evoked impressions of opposite-direction motion, but the dynamic MAE proved somewhat stronger for phantom than control inducers. Also, MAEs were more strongly modulated by the physical contrast of the control inducers and less influenced by the contrast of the phantom inducers. These findings suggest that low-level motion adaptation strongly contributes to the MAEs elicited in the gap region but that the magnitude of adaptation is further modulated by phantom perception.

## Introduction

**Figure 1.**
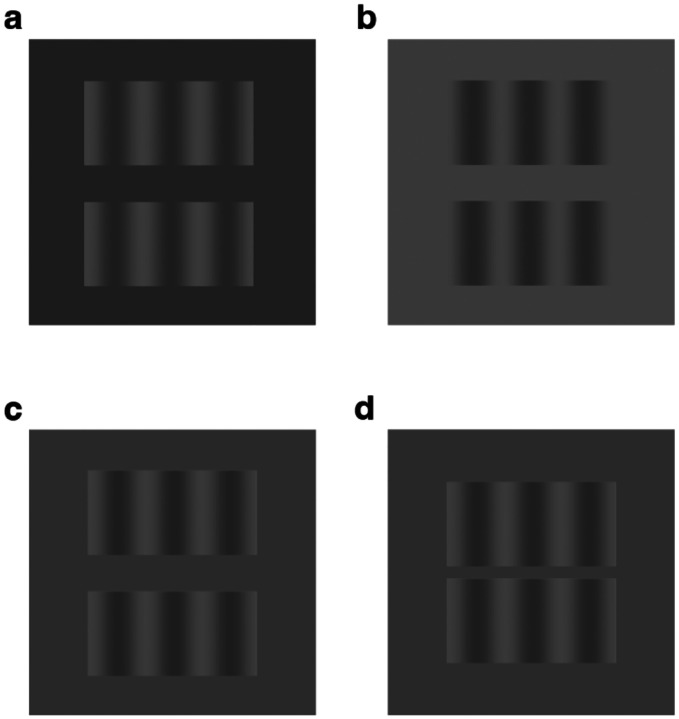
Examples of visual phantom inducers and phantom-control inducers. **a.** Example of dim phantoms: The luminance within the blank gap region matches that of the darkest portions of the grating inducers, eliciting the impression of dim phantom stripes that extend through the gap for most observers. **b.** Light phantoms: The luminance of the blank gap region matches that of the light portions of the inducers, eliciting the impression of light phantom stripes that extend through the uniform gap. **c.** Phantom-control gratings: the luminance within the blank gap matches the mean luminance of the grating inducers, eliciting a weaker impression of an opposite-phase pattern in the gap. **d.** Phantom-control gratings with a narrower gap between grating inducers, leading to a stronger impression of an opposite-phase pattern. Note that identical grating inducers are shown in all four plots.

The constructive nature of the visual system can be demonstrated by perceptual filling-in phenomena of which there are many [1]. One of the most striking is phantom visual motion, first described by Tynan and Sekuler [2]. This illusion can be experienced when two low-contrast collinear grating inducers, separated by a blank gap (see Figure 1a), drift in phase – thereby creating the illusory impression of visual motion within the uncontoured gap region [3, 4].

Multiple studies have led researchers to propose that the visual phantom illusion arises from an active filling-in process that occurs at early stages of visual processing. One key finding was that prolonged viewing of moving phantom inducers can lead to a motion aftereffect (MAE) when a static test pattern is subsequently presented in the blank gap region [3]. This MAE was interpreted to suggest that the neural representation of the visual phantom is capable of adapting local motion detectors at an early stage of the visual pathway. Other psychophysical studies have shown that when grating inducers are presented dichoptically (i.e., upper and lower inducers presented to separate eyes), visual phantoms are still experienced, implying that the visual phantom illusion is generated at a processing stage after binocular combination [2]. In addition, visual phantoms require considerably more time for perceptual completion if the size of the gap between the inducers is increased, suggesting the involvement of an active filling-in process [2, 5]. Research has further shown that phantom filling-in is abolished during spontaneous binocular rivalry alternations and also when flash suppression is used to render the inducers phenomenally invisible [5]. These findings have led researchers to propose that rivalry suppression likely occurs at an earlier processing stage than phantom filling-in. Human neuroimaging studies have also provided support for the active filling-in hypothesis, showing that collinear visual phantom inducers evoke enhanced fMRI responses in V1 and V2 regions that correspond to the blank gap between the inducers [6]. Interestingly, this study showed that perception of the visual phantom illusion under conditions of binocular rivalry led to strongly correlated modulations in these early visual areas, thereby linking neural activity to conscious perception [6].

Despite the positive evidence supporting an active filling-in account of the visual phantom illusion, it remains possible that some of the purported effects described above may reflect low-level mechanisms that operate independently of the perception of visual phantoms *per se*. For example, low spatial frequency gratings are typically required to generate visual phantoms that can span gaps exceeding more than a degree of visual angle, while other work has shown that drifting gratings are more likely to induce a motion aftereffect in immediately adjacent regions of the visual field if the stimuli are presented at a low spatial frequency (i.e., less that 1 cpd) [7]. Such requirements for successful adaptation transfer to neighboring spatial regions are consistent with the predictions of low-level motion energy models [8].

The goal of our study was to test whether the vividness of visual phantom perception has an impact on the resulting MAE while controlling for the low-level motion energy signals arising from the inducer gratings. Phantom perception is known to depend on the relationship between the luminance of the gratings and the luminance of the background. For example, if the darkest portion of the phantom-inducing gratings matches the background luminance, then one typically perceives the dark portions of the inducers extending through the gap region (Figure 1a). If instead, the lightest portion of the gratings matches the background, then one typically perceives those lighter portions extending through the gap (Figure 1b). Such effects of phantom filling-in have been attributed to an inference of perceptual transparency made by the visual system, as the respective dark or light phantoms described above evoke the impression of cloud-like structures that appear figural or positioned in front of the intervening gap [9].

Such phantom inducers can be distinguished from the situation in which the mean luminance of the inducer gratings matches that of the background or what we will refer to as phantom-control inducers. At low to moderate contrast levels, such control inducers typically elicit a weaker impression in the gap region (Figure 1c), and notably, the impressions of brightness modulation in the gap are opposite in spatial phase when compared with the inducers.

When stimulus contrast is manipulated, further differences can be observed between phantom inducers and control inducers. Control inducers tend to evoke stronger impressions of a contrast-reversed pattern in the gap region if they are presented at full contrast, a phenomenon sometimes described as grating induction [10], which has been attributed to the same low-level mechanisms involved in simultaneous brightness contrast [11]. With control inducers of moderate contrast, it is still possible to elicit a vivid impression of an opposite-phase pattern if the gap between the inducers is made extremely narrow (Figure 1d), a finding that is consistent with local center-surround inhibition, possibly emerging at the level of the retina [12]. By comparison, visual phantoms appear quite vivid when the inducers are presented at low to moderate contrast levels, even across gaps exceeding 1 degree of visual angle.

In the present study, we tested whether phantom inducers evoke stronger motion aftereffects than control inducers when both are presented with the same physical contrast, thereby controlling for low-level effects of motion adaptation. We assumed that if the vividness of the visual phantoms is related to the strength of the resulting motion aftereffect, this would imply that the motion processing system receives input from early visual areas responsible for the generation of moving visual phantoms. However, if phantom inducers were to elicit higher ratings of vividness yet fail to evoke a stronger MAE in comparison to the control inducers, such a dissociation would imply that separate mechanisms underlie motion adaptation and the perceptual interpretation of phantom-inducing stimuli.

The original studies of the phantom motion aftereffect evaluated the MAE by presenting static grating patterns at test [3] or what is commonly referred to as the static MAE (sMAE). In the present study, we investigated the consequences of motion adaptation by using both static and dynamic test patterns. It is well documented that dynamic test stimuli, such as a counterphasing sinewave grating pattern that contains opposing directions of motion energy, are sensitive to higher-level forms of motion adaptation. For example, if observers are adapted to monocular drifting motion at a specific visual location in one eye, then the presentation of a dynamic test in the corresponding location of the other eye will lead to a motion aftereffect whereas a static test stimulus will not [13, 14]. Such findings have led to the proposal that perception of the dynamic motion aftereffect (dMAE) involves higher-level visual neurons in binocular motion-processing areas, whereas the sMAE occurs at an earlier processing stage that likely involves monocular neurons in V1. Thus, if the visual phantom illusion primarily engages higher-level visual mechanisms, one might reasonably expect to find a stronger dMAE than sMAE after adaptation to phantom visual motion.

In the present study, we evaluated the perception of both static and dynamic test stimuli after observers were adapted with phantom-inducing gratings or phantom-control gratings that drifted in a single direction for a prolonged period (see Methods). The inducers were presented at either low, medium or high contrast (7.5%, 15% or 60%, respectively) to evaluate the contrast dependence of the aftereffects, and the test patterns were always presented in the visual location corresponding to the gap region between the inducers. We hypothesized that if the neural representation of visual phantoms provides direct input to the motion processing system, then the phantom inducers should elicit a stronger MAE than the control inducers. Moreover, differences in MAE strength between phantom and control inducers might be magnified by using dynamic test stimuli if one assumes that the moving phantom illusion involves a higher-level form of motion processing. In addition, MAEs in the control condition might prove to be more sensitive to low-level manipulations of inducer contrast than the phantom condition, assuming that the former is more stimulus-driven and dependent on low-level visual processes. Alternatively, if the control inducers were to elicit equally strong MAEs as the phantom inducers, this would suggest that contrary to previous claims, phantom-inducing gratings lead to the activation of low-level motion detectors via mechanisms that are largely independent of the illusory percepts that they induce.

We recruited 20 participants in this study to evaluate the strength of the MAEs elicited by phantom-inducing gratings, phantom-control gratings, and also a full-grating control condition to provide a ceiling measure of MAE strength. We evaluated the static and dynamic MAEs in separate experimental sessions (60–90 minutes each), with order counterbalanced across participants. Participants were required to exhibit at least 50% MAE bias in a full-grating adaptation condition for inclusion in our analysis pipeline; thus, we included 18 out of 20 participants in our final analyses.

## Results for static MAE

In the static MAE experiment, participants first rated the subjective vividness of their impressions in the blank gap region while viewing drifting phantom-inducing or phantom-control gratings at each of 3 contrast levels (see Methods). Examples of these displays can be seen in Figure 1a and 1c. The vertically oriented inducer gratings (spatial frequency of 0.25 cycles per degree) spanned 12° × 6° each, separated by a horizontal gap (12° × 2.6°). Then, participants performed the static MAE task. Each trial started with a 45-s adaptation phase during which a pair of gratings drifting leftward or rightward at 1.5 Hz. Immediately following this adaptation phase, the inducers were removed and a static test grating of matching spatial frequency (7.5% contrast) was presented in the blank gap region (12° × 2.6°). Observers pressed 1 of 3 keys to indicate whether they perceived leftward motion, rightward motion, or no motion. If illusory motion was experienced, the observer maintained the keypress response for the duration of the experience of illusory motion.

**Figure 2.**
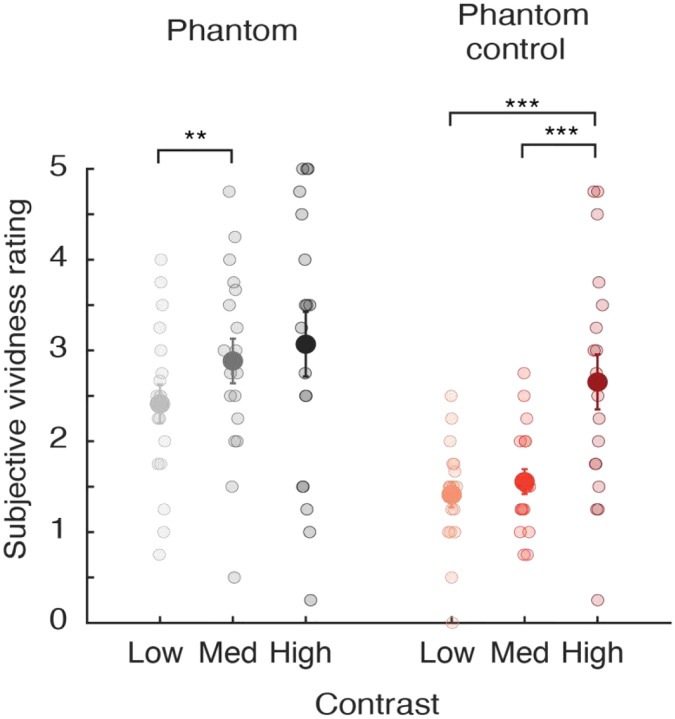
Subjective vividness ratings for phantom-inducing and phantom-control gratings in the static MAE experiment (1.5 Hz drifting speed, N = 18). Each participant rated, on a scale from 0 to 5, the vividness of their impressions in the gap region between the inducer gratings, which were presented at low, medium or high contrast. Paired t-tests: phantom low versus phantom control low: *t*(17) = 3.47, *p* = 0.0030; phantom medium versus phantom control medium: *t*(17) = 4.46, *p* = 0.00035; phantom high versus phantom control high: *t*(17) = 0.95, *p* = 0.36. Larger symbols with error bars depict mean vividness ratings with ±1 standard error of the mean (s.e.m.). Smaller symbols show the vividness ratings of individual participants. Paired t-tests: * p < 0.05, ** p < 0.01, *** p < 0.001.

As can be seen in Figure 2, observers reported that the phantom-inducing gratings led to more vivid percepts in the blank gap region than the phantom-control condition, and these differences appeared more prominent at low and medium contrast levels. For the phantom-inducing gratings, most participants reported perceiving visual phantoms that extended through the entire gap region with an average rating greater than 2 (see Table 1), while many participants reported that the phantom control condition led to an incomplete or disconnected percept in the gap region at low and medium contrasts with average vividness ratings lower than 2. A repeated-measures ANOVA revealed a significant difference between the two inducer types (*F*(1, 17) = 11.13, *p* = 0.004), with a significant main effect of inducer contrast (*F*(2, 34) = 11.65, *p* = 0.0001), and a marginally significant trend of an interaction effect between inducer type and stimulus contrast (*F*(2, 34) = 3.06, *p* = 0.06). This trend towards an interaction effect was due to the significant increase in vividness ratings from low to medium contrast for the phantom inducers (*t*(17) = 3.15, *p* = 0.0058), whereas vividness significantly increased between medium and high contrast levels for the control inducers (*t*(17) = 4.37, *p* = 0.0004). The former finding is consistent with the general claim that visual phantoms tend to be most vivid if the inducing stimuli are presented at a fairly low contrast (typically, 15–25%) [2, 6, 15]. In comparison, the control inducers elicited the strongest impressions in the gap region when presented at high stimulus contrast, due to the presumed effects of grating induction or simultaneous brightness contrast [10, 16].

In the static MAE task, motion reports were coded with respect to the adapting motion direction, and we quantified the proportion of trials with reports of opposite-direction motion, same-direction motion, or no clear motion percept. Same direction reports were extremely rare (less than 5% on average for all conditions), while reports of no motion occurred quite often in the visual phantom and phantom control conditions (ranging from 10 to 40% of all trials).

**Figure 3.**
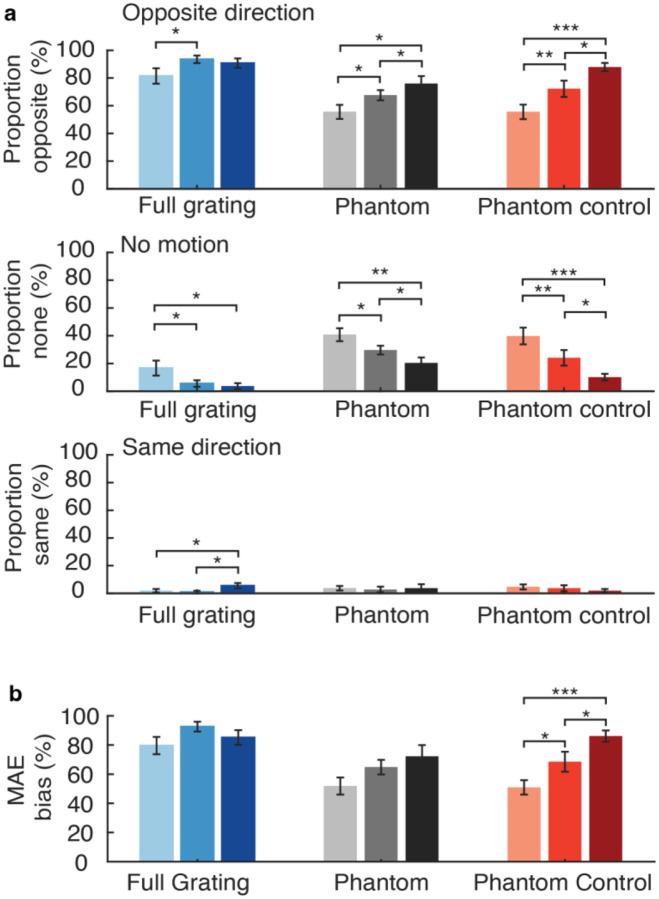
Motion perception reports for static test grating and percentage sMAE bias (N = 18). **a.** Bar plots show proportion of opposite direction, no motion, and same direction reports for each experimental condition and contrast level (darker colors indicate higher contrast). **b.** MAE bias index plotted by experimental condition and contrast level, based on percentage of opposite-direction responses minus same-direction responses. Error bars represent ± 1 s.e.m. Paired t-tests: * p < 0.05, ** p < 0.01, *** p < 0.001.

Opposite-direction motion was perceived most often in the full grating condition, with reports exceeding 90% in the medium and high contrast conditions (see Figure 3a). This was to be expected given that the full grating adaptor spanned the gap and provided direct stimulation at that visual location, leading to a strong sense of illusory motion when the static test grating was presented within the horizontal ‘gap’ portion of the display. In comparison, illusory opposite-direction motion occurred less often in the phantom and phantom-control conditions, but with about equal prevalence for both, ranging from ~50% to ~80% occurrence depending on the contrast level.

We then calculated percent MAE bias, which reflected the percentage of opposite-direction motion reports minus the percentage of same-direction reports for each condition. Here, bias values exceeding 0% indicate a directional bias consistent with the motion aftereffect. We found that phantom-inducing gratings led to a consistent MAE bias for all contrast conditions, but these effects were no stronger, on average, than those observed in the phantom-control condition (see Figure 3b). In addition, we found that sMAE bias tended to increase as a function of inducer contrast for both the phantom and control conditions. However, this graded increase was more clearly evident in the phantom-control condition, with significant increases in MAE bias observed between low and medium inducer contrasts (*t*(17) = −2.82, *p* = 0.012) and also between medium and high contrasts (*t*(17) = −2.82, *p* = 0.012; note, while atypical, we confirmed these separate tests led to the same t-value score). In comparison, manipulations of the contrast of the phantom-inducing gratings did not lead to significant changes in MAE bias (phantom low-contrast vs. medium contrast, *t*(17) = −1.77, *p* = 0.095, phantom medium versus phantom high *t*(17) = −1.81, *p* = 0.088). Although we did not find evidence of a significant interaction effect between inducer type and contrast level for percent MAE bias (*F*(2,34) = 1.50, *p* = 0.24), our subsequent analyses of the static MAE duration did provide positive evidence of such an interaction as indicated below.

**Figure 4.**
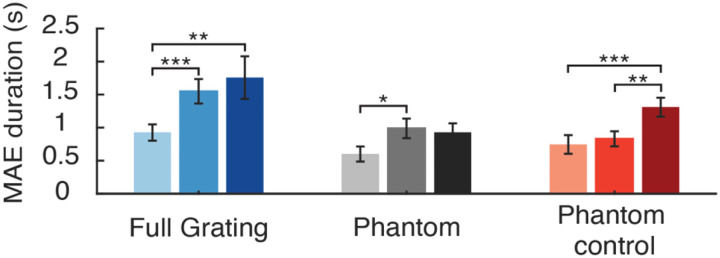
Average MAE duration in the static MAE experiment (N = 18). Paired t-tests results: Phantom low versus phantom medium: *t*(17) = −2.61, *p* = 0.018, phantom low versus phantom high: *t*(17) = −1.67, *p* = 0.11; phantom medium versus phantom high, *t*(17) = 0.34, *p* = 0.74; phantom control low versus phantom control medium, *t*(17) = −0.62, *p* = 0.54; phantom control low versus phantom control high, *t*(17) = −4.81, *p* = 0.0002; phantom control medium versus phantom control high, *t*(17) = −3.80 *p* = 0.0014.

We performed a similar analysis of the mean duration of sMAE reports, using the actual duration of responses for opposite-direction reports, and coding responses of same-direction and no motion responses as 0 seconds before calculating the average duration for each condition. As expected, sMAE durations were higher in the full-field grating condition, and sMAE durations increased as a function of stimulus contrast (see Figure 4).

We conducted a repeated-measures ANOVA to directly compare the phantom and phantom-control conditions. This analysis revealed a marginally significant effect of inducer type (*F*(1, 17) = 3.22, *p* = 0.091), a significant effect of stimulus contrast (*F*(2, 34) = 6.47, *p* = 0.004), and a significant interaction effect between inducer type and stimulus contrast (*F*(2, 34) = 4.42, *p* = 0.02). The interaction effect was of particular interest, as it appeared attributable to the fact that MAE durations for the phantom inducers significantly increased between the low and medium contrast conditions (*t*(17) = 2.61, *p* = 0.018), whereas for the phantom-control inducers, a significant increase in MAE duration was only observed between the medium and high contrast conditions (*t*(17) = 3.32, *p* = 0.004). These differential trends were in general agreement with subjective vividness ratings (see Figure 2), which revealed a significant increase in vividness ratings for phantom inducers between low and medium contrast conditions, while for the phantom-control inducers a significant increase was observed between medium and high contrast conditions.

As an interim summary, we found that phantom-inducing gratings evoked a static MAE, consistent with previous reports [3]. However, we further observed that phantom-control inducers led to static MAE bias effects of similar strength, suggesting that a considerable proportion of the MAE elicited by both types of inducers may be attributable to low-level effects of motion adaptation. Even at low and medium contrast levels, for which observers described perceiving more vivid impressions in the gap region in the phantom condition than in the control, sMAE bias effects proved no stronger for the phantom inducers when compared to the control. That said, the magnitude of the sMAE bias effect was highly dependent on stimulus contrast in the control condition and showed less evidence of dependency on contrast in the phantom condition. These findings were bolstered by our analyses of sMAE duration, which revealed a significant interaction effect between the two inducer types and stimulus contrast. It is noteworthy that the duration of the sMAE increased between low and medium stimulus contrasts (7.5% vs. 15%) in the phantom inducer condition, in general agreement with the observed increase in subjective vividness ratings (see Figure 2), whereas sMAE durations significantly increased between the medium and high contrast conditions (15% vs. 60%) for the phantom control, again in agreement with the increase in reported vividness between these particular conditions. Taken together, our findings suggest that while a considerable proportion of the static MAE elicited by phantom-inducing gratings can be attributed to low-level effects of adaptation, the strength or duration of the sMAE is further modulated by perceptual attributes of the visual phantom illusion.

## Results for dMAE

At the beginning of the dMAE experimental session, we also had observers report the vividness of their impressions in the blank gap region when presented with drifting phantom-inducing gratings and phantom-control gratings at various contrast levels. A somewhat higher temporal frequency was used for both adaptation and test in the dMAE experiment, as our pilot work indicated that full-field gratings led to a more consistent dMAE if the counterphasing test stimulus was presented at 2Hz rather than 1.5 Hz.

**Figure 5.**
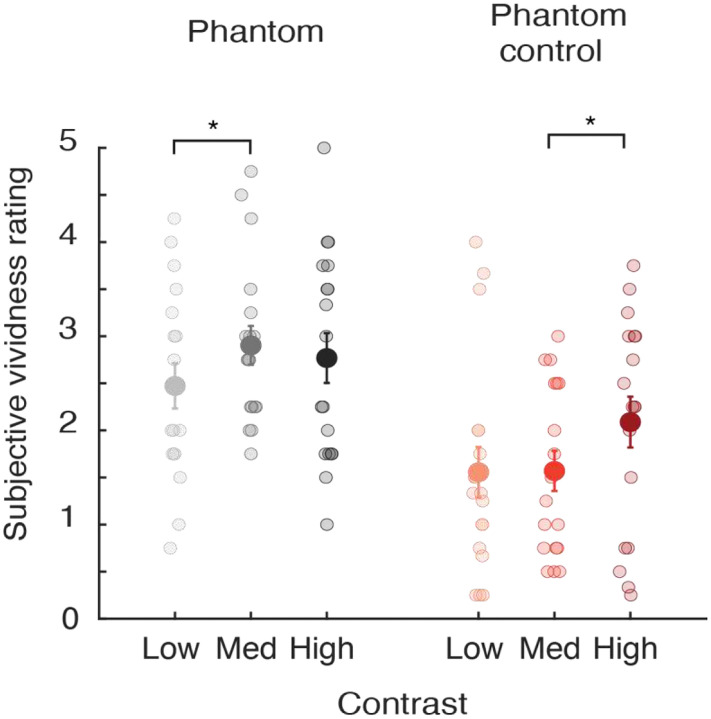
Subjective vividness ratings in the dynamic MAE experiment (2 Hz drifting speed, N = 18). Each participant rated, on a scale from 0 to 5, the vividness of their impressions in the gap region between the inducer gratings, which were presented at low, medium or high contrast of 7.5%, 15% or 60%, respectively (see Methods). Paired t-tests: phantom low versus phantom control low: *t*(17) = 2.37, *p* = 0.030; phantom medium versus phantom control medium: *t*(17) = 3.78, *p* = 0.0015; phantom high versus phantom control high: *t*(17) = 1.83, *p* = 0.085.

As expected, vividness ratings were higher overall in the phantom condition as compared to the phantom control (see Figure 5). In addition, vividness increased significantly between the low contrast and medium contrast conditions for phantom-inducing gratings (7.5% vs. 15%) (paired samples t-test: *t*(17) = −2.73, *p* = 0.014), whereas vividness increased between the medium and high contrast conditions for the phantom-control inducers (paired samples t-test: *t*(17) = −2.16, *p* = 0.045). The pattern of results for these vividness measures proved to be very similar to those observed in Figure 2, and consistent with prior work showing that visual phantoms attain a maximal saliency with inducers of fairly low contrast [2, 6], whereas phantom-control inducers elicit the strongest impressions in the gap region if they are presented at high contrast [7, 10].

**Figure 6.**
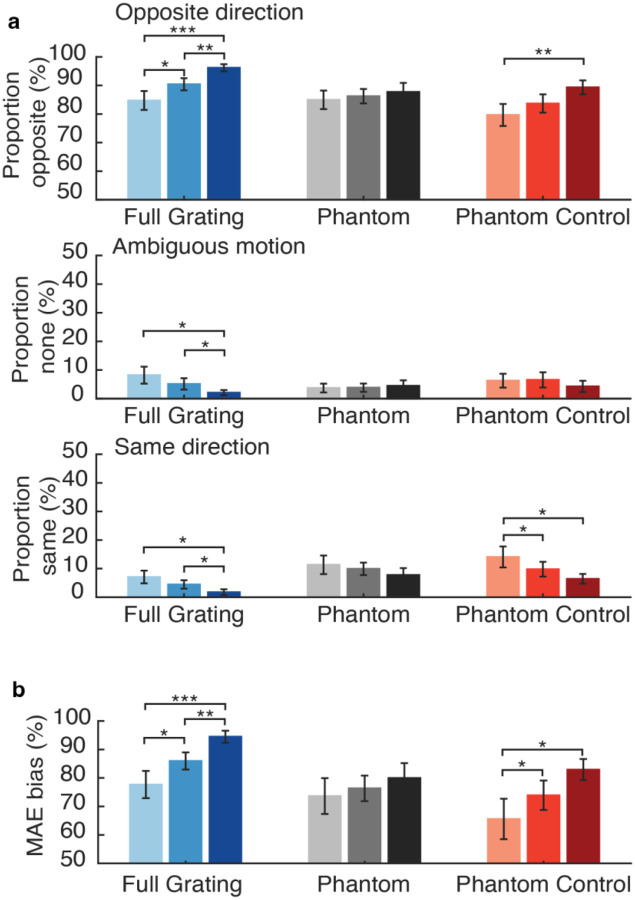
Motion perception reports and percentage MAE bias in the dynamic MAE experiment (N = 18). **a.** Proportion of each percept across experimental conditions plotted by contrast level (darker colors, higher contrast). **b.** Percent dMAE bias index. Paired t-tests with * p < 0.05, ** p < 0.01, *** p < 0.001 indicate statistically reliable differences in MAE bias between contrast levels.

In the main dMAE experiment, participants viewed visual phantom inducers or control inducers that drifted leftward or rightward at 2 Hz for an 8-s adaptation phase, after which a dynamic counterphasing grating (7.5% contrast) was presented in the gap region for 1 second. Participants reported whether the dynamic test stimulus appeared to be moving leftward, rightward, or whether its perceived motion was ambiguous. As shown in Figure 6a, the opposite-direction reports increased in a graded manner with contrast and was generally higher in the full-field grating condition than in the phantom or phantom-control conditions. In the phantom condition, the opposite-direction reports remained quite stable across contrast levels, while they clearly rose with increasing contrast in the phantom-control condition.

Next, we calculated percent dMAE bias based on the percentage of opposite-direction minus same-direction reports (see Methods). As can be seen in Figure 6b, the dMAE bias was strongest overall in the full grating condition and increased significantly at higher stimulus contrast, as was indicated by paired t-test comparisons.

We conducted a repeated-measures ANOVA to evaluate the magnitude of dMAE bias in the phantom and phantom-control conditions across manipulations of contrast. This analysis revealed a significant main effect of inducer type (*F*(1, 17) = 6.57, *p* = 0.020) and a significant main effect of contrast (*F*(2, 34) = 5.64, *p* = 0.008), while the interaction effect between inducer type and stimulus contrast was not statistically significant (*F*(2, 34) = 1.29, *p* = 0.29). While the increase in dMAE bias at higher inducer contrasts was expected for stimulus-driven reasons, the fact that dMAE bias was significantly greater for the phantom condition than the phantom control suggests that visual phantom perception does contribute to the motion aftereffect. Planned comparisons further revealed that for the low contrast condition, phantom inducers led to significantly stronger dMAE bias than the phantom-control inducers (*t*(17) = 2.92, *p* = 0.0095).

Although the interaction effect between inducer type and stimulus contrast was not reliable, we did find that increasing the contrast of the control inducers led to significant increases in dMAE bias, whereas increasing the contrast of the phantom inducers did not lead to statistically reliable changes in dMAE bias. This pattern of results was generally consistent with those observed for the static MAE (cf. Figure 3b), where again, contrast manipulations had a more reliable impact on MAE bias in the phantom-control condition. One interpretation of these trends across both experiments is that contrast manipulations have a powerful impact on the magnitude of low-level adaptation that is elicited by the phantom-control inducers whose mean luminance matches the background. In comparison, perceptual processing of the phantom-inducing gratings is generally more stable or invariant to manipulations of stimulus contrast.

## Discussion

In this project, we investigated whether visual phantoms can induce a motion aftereffect while controlling for low-level effects of motion adaptation, and further compared the magnitude of static and dynamic MAEs elicited by visual phantoms and phantom-control stimuli. While full-field grating displays led to the strongest MAEs, both phantom-inducing and phantom-control gratings led to reliable motion bias effects, consistent with the notion that low-level mechanisms of motion adaptation were involved in both cases. However, we also found that dMAE bias was significantly stronger for phantom inducers than for phantom-control gratings, with evidence of a clear difference found at low contrast. Moreover, manipulations of stimulus contrast had a more consistent impact on the magnitude of the static and dynamic MAEs elicited by the phantom-control inducers. In comparison, the MAEs elicited by phantom inducers were generally more stable or invariant to contrast, perhaps reflecting the fact that visual phantoms are perceived as quite vivid even when the phantom inducers are presented at a low stimulus contrast. Taken together, these findings indicate that the visual phantom illusion does contribute to both types of motion aftereffects.

**Figure 7.**
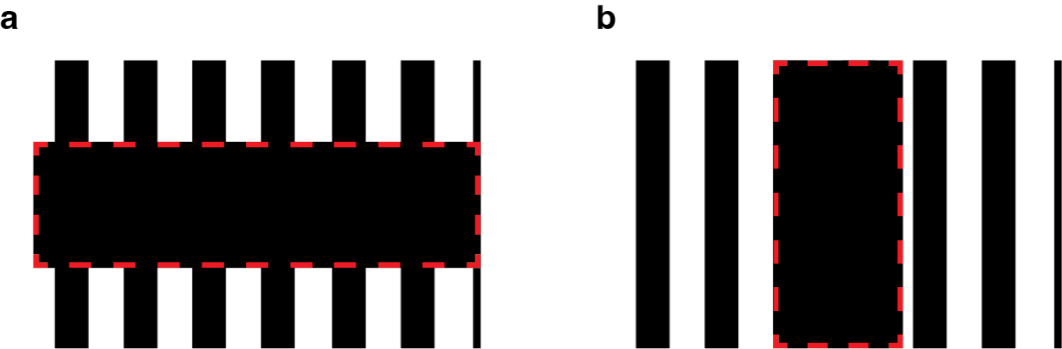
Illustration of phantom-inducing and phantom-control conditions from Weisstein et al., 1977 [4]. **a.** Phantom condition. Vertical collinear square-wave gratings are separated by a horizontally elongated blank gap. The grating inducers drifted from left to right. **b.** No-phantom control condition. Two vertical gratings are separated by a vertical gap region. This configuration lacks collinearity between the two vertical gratings, and the vertical components of the gratings do not terminate at the edge of the gap region. Dashed red rectangular region indicates location of the blank gap in the two displays.

A previous study by Weisstein et al. (1977) reported that visual phantoms could induce a very strong sMAE whereas a no-phantom control condition did not, but that control condition relied on a very different spatial arrangement of inducers [3]. Specifically, the phantom condition consisted of two vertically oriented square-wave gratings separated by a horizontal gap (Figure 7a), similar to the current study, whereas the control display consisted of vertical square-wave gratings positioned to the left and right of a vertical gap (Figure 7b). When these gratings drifted leftward or rightward, the differences in stimulus configuration across conditions could have led to differential stimulation of low-level motion energy detectors. For example, if one assumes a motion energy model that resembles V1 direction-selective complex cells [8] with some unit receptive fields that span the gap region and neighboring surround, then the drifting gratings in the phantom condition would be expected to stimulate these direction-selective units to a much greater extent than those in the control condition. In our study, the phantom-inducing gratings and the control gratings shared the same spatial arrangement and thereby avoided this potential confound. Such differences between studies likely explain why Weisstein et al. failed to observe a reliable sMAE with their control inducers [3], whereas our phantom-control inducers led to a prominent sMAE. Thus, the present study provides more rigorous evidence to support the assertion that phantom perception contributes, at least to some extent, to motion adaptation and the motion aftereffect.

Our findings provide support for the notion that visual phantom perception involves an active filling-in process [1, 2, 5] that operates at early stages of cortical visual processing [6]. Phantom perception can be distinguished from the phenomenon of grating induction, which can be observed when collinear gratings separated by a narrow gap are presented on a mean luminance background; such viewing conditions lead to the perception of a salient opposite-phase pattern in the intervening gap region (e.g., Figure 1d). The phenomenon of grating induction can be maximized if the inducing gratings are low spatial frequency (less than 1 cpd) and high contrast (typically 75–100%), and if the gap between the inducers is narrowed to just fractions of a degree of visual angle [10]. Under such optimal conditions of grating induction, moving grating inducers can induce a static MAE in the gap location that is just as strong or stronger than that evoked by a full-field drifting grating [7]. Here, we presented drifting phantom-control inducers with a much larger gap size of 2.6° and found that these stimuli could evoke a reliable MAE in the gap region, though it was considerably weaker than that evoked by full-field stimulation. Nevertheless, we found that the MAE elicited by phantom-control gratings proved strongest at high stimulus contrast, consistent the notion that grating induction arises from contrast-sensitive mechanisms of lateral inhibition with contributions likely originating as early as the retina [12].

Although some have argued that visual phantom perception might arise from a common mechanism as grating induction [16], a more prominent view is that phantom perception arises from the perceptual inference of transparency [15, 17]. Specifically, the lack of luminance boundaries between portions of the phantom-inducing gratings and the intervening gap (e.g., Figures 1a and 1b) encourages the visual system to infer the presence of semi-transparent ‘stripes’ extending across the blank gap region. Along these lines, our finding that visual phantom perception can modulate the strength of the MAE provides further evidence of the types of form-motion interactions that can take place in the visual system [18]. Other work has shown that when two drifting gratings with different trajectories are superimposed (and presented within a circular aperture), one typically perceives a coherent plaid pattern that moves along a single intermediate trajectory [19]. However, such coherent perception can be fractionated if cues of visual transparency are introduced, which facilitates perception of the two separate component motions [20]. Another type of form-motion interaction can be observed in the well-known barber-pole illusion, which demonstrates how local motion cues can be overridden by global cues to infer the global motion direction [21]. For example, if one were to view a vertical grating drifting rightward within a diamond-shaped aperture, the perceived motion trajectory can be modified to convey either 45° upward or downward motion, depending on which border of the diamond aperture appears to be positioned in front of the drifting grating (via stereo-depth cues). In effect, the drifting grating will appear to be sliding toward (and underneath) the edge-border that lies in front of the grating. In this way, cues about visual form can strongly influence the perception of global motion. Investigations of the motion aftereffect elicited by such visual displays have revealed that the MAE reflects a weighted combination of the local motion signals and the perceived global motion [22]. If one considers visual phantom perception as arising from a form-based interpretation of transparency, then the modulatory effects we observe for phantom inducers, relative to the phantom control, can also be interpreted in terms of the relative weighting of low-level motion cues and implied form cues. In summary, our findings suggest that illusory impressions of visual form, as exemplified by the moving phantom illusion, are indeed processed by the human motion system, while low-level motion signals provide the primary inputs to drive the adaptation of this system.

## Methods

### Participants

We recruited 20 participants (7 males, 13 females), aged 18–34 (mean = 23.9 ± 5.9) for this study. Participants were recruited through the Vanderbilt University Psychology Department website or through word of mouth. All had normal or corrected-to-normal visual acuity, and none had any history of neurological disorder. The experiment was performed in two sessions, each session lasting 60–90 minutes depending on the duration of voluntary rest periods. The static MAE task was performed in one session and the dynamic MAE task was performed in the other session, with the order of sessions counterbalanced across participants. Observers were compensated at a rate of $15/hour for their participation. All participants provided informed consent, and the study was approved by the Vanderbilt University Institutional Review Board. All experiments were performed in accordance with the Code of Ethics of the Declaration of Helsinki.

### Experimental Setup

The experiments were implemented using MATLAB and Psychophysics Toolbox [23–25] on a Mac Pro computer running under the OS X El Capitan (version 10.11.6) operating system. The visual stimuli were displayed on a CRT monitor (DELL-06D251, 40 cm width × 30 cm height) whose resolution was set at 1280 × 1024 and frame rate set at 85 Hz. The display subtended 47° × 35.25° at a viewing distance of 46 cm. The luminance output of the CRT was calibrated and linearized using a Minolta CS-100A colorimeter. The participant’s head position was stabilized using a chinrest.

### Visual Stimuli

For both static and dynamic MAE experiments, observers were adapted with 3 different grating conditions (full grating, phantom inducer, and phantom control) presented at each of 3 contrast levels (7.5%, 15%, and 60%). Both the phantom-inducing gratings and the control gratings were presented with the same spatial arrangement, involving a pair of 6° high × 12° wide vertical grating inducers (0.25 cyc/deg spatial frequency) separated by a 2.6° horizontal blank gap (see Figure 1a and 1c) on a uniform background with a luminance of 73.6 cd/m^2^. Thus, there were 9 experimental conditions in all. Each trial began by having the participant fixate a central fixation cross (0.45° × 0.45°) and then press the space bar to begin stimulus presentation. The gratings drifted either leftward or rightward (counterbalanced across trials). Immediately after this adaptation period, a test grating appeared on the screen (size 1.73° high × 12° wide grating with 7.5% contrast and 0.25 cyc/deg spatial frequency), occupying a central, thinner part of the blank gap region.

The phantom and phantom-control conditions were as follows. In the phantom condition, the luminance of the gratings’ dark contours matched the background luminance, reliably producing a vivid impression of visual phantoms. In the phantom-control condition, the space-averaged luminance of the grating matched that of the background luminance, a condition producing only a faint impression of a phantom. In addition, we presented full-field gratings with the darkest portions of the gratings matching the background luminance to test the effects of local stimulus adaptation at the visual location where the subsequently viewed test grating appeared. We expected that this condition would evoke strong MAEs (which it did) and thereby provide a benchmark for comparison with that resulting from adaptation in the phantom and phantom-control conditions.

### Perceptual Vividness Rating Task

Prior to each of two experimental sessions, participants were instructed to report on the vividness of their impressions in the gap region for separate presentations of the phantom-inducing gratings and the phantom-control gratings, which drifted unidirectionally leftward or rightward at the same rate as the main experiment. A pair of 6° high by 12° wide grating inducers, separated by a 2.6° gap, was displayed on one side of the screen, and on the other side was displayed a 3% contrast reference grating (2.6° high by 12° wide 3%). The reference grating drifted in the same direction as the inducer pair. The placement of the pair of inducers and the reference grating was randomly alternated between left and right side of the display from trial to trial. Participants could freely look back and forth between the inducer gratings and the reference grating to make their perceptual assessment, and were asked to rate the vividness of their impressions in the gap region between the inducers on a scale from 0 to 5 (see Table 1).

Each inducer type was presented at low, medium and high contrast in a randomized order, and a total of 4 rating reports were obtained in each condition. Within each inducer type by contrast condition, the gratings drifted either rightward or leftward (2 trials each). The motion drift rate for both the inducer pair and the reference grating was 1.5 Hz for the static MAE session and 2 Hz for the dynamic MAE session, matching the drifting speed of the main tasks, respectively.

### Static MAE Task

On each trial of the sMAE experiment, observers were instructed to maintain fixation while viewing either full-field, phantom-inducing or phantom-control gratings that drifted leftward or rightward (temporal frequency, 1.5 Hz) for a 45-second adaptation period. The adapting gratings could appear at one of three contrast levels (7.5%, 15%, or 60%). Immediately afterward, a static test grating (7.5% contrast) was centrally presented within the visual location that corresponded to the gap region for the phantom and phantom-control conditions. Observers used 1 of 3 keys to report their perception of the static test. If the observer experienced either leftward or rightward motion, they were instructed to hold down the corresponding key until that impression of motion dissipated. If there was no impression of motion on a given trial, participants were instructed to press a third key to indicate the lack of a motion percept. As soon as the key was released, the test stimulus disappeared, and the participant was free to advance to the next trial. The visual display included random line segments in the far periphery (beyond the inducer stimuli) to provide a reference frame for evaluating the direction and duration of the sMAE. Participants performed 6 trials per condition, with 3 leftward-motion and 3 rightward-motion trials, for a total of 54 trials in the experimental session. The order of all conditions was randomized across trials.

### Dynamic MAE Task

In the dynamic MAE experiment, the inducing gratings drifted at a speed of 2 Hz for adaptation period of 8s, after which a dynamic counterphasing test grating appeared on the screen for 1s, followed by a 3-s blank period to allow for the participant to respond. The participant pressed one of three keys to indicate whether the dynamic test elicited an impression of leftward motion, rightward motion, or no clear motion direction. The test stimulus for the dMAE consisted of a counterphasing grating with contrast that changed sinusoidally at a rate of 2 Hz (maximum contrast, 7.5% amplitude).

The experimental conditions consisted of adaptation to full-field drifting gratings, phantom-inducing gratings, and phantom-control gratings, with each grating type presented at each of the 3 contrast levels. The experiment consisted of 12 trials of leftward motion and 12 trials of rightward motion for each grating type by contrast condition, resulting in 24 trials for each condition and a total of 216 trials for the dMAE experiment. The order of conditions was randomized across trials. After every 10 trials, a 10-s break was included to minimize effects of fatigue.

### Analyses

Responses were classified as *opposite* if the perceived test direction was the opposite that of the adaptation stimulus, *none* if there was no motion perceived or if perception was ambiguous, and *same* if the perceived direction of the test stimulus was the same as that of the adapting stimulus. A motion aftereffect bias score was then calculated, by scoring the number of responses for all trials within each condition as 1 if the response was *opposite*, 0 if the response was *none*, and −1 if the response was *same*. All of these scores were then summed together, divided by the total number of trials within each condition, and converted to a percentage score, leading to our motion aftereffect bias measure.

To ensure that participants were performing the task reliably and exhibiting typical effects of motion adaptation, a criterion was established that required that participants exhibit a minimum of 50% MAE bias averaged across all three full-field grating conditions for inclusion in the subsequent analyses. Among the recruited participants, 18 of 20 attained this criterion, and those data are reported in this study.

We also analyzed the average MAE duration in the sMAE experiment. Here, all “same” and “none” responses were considered to be 0 seconds in duration, and the average sMAE duration was calculated across trials within each condition.

Within-subjects ANOVA models were fitted to the data using R (version 4.4.0) to test for within-subjects effects and interaction effects between inducer type and contrast level.

## Figures and Tables

**Table 1.** Vividness rating scale provided to observers to evaluate their impressions in the blank gap region during presentation of phantom-inducing gratings and phantom-control gratings. Observers were instructed to rate the subjective vividness of their impressions in comparison with a 3% contrast reference grating that was concurrently displayed.

Score	Perceived vividness
**0**	No pattern perceived at all
**1**	Very faint impression of a grating pattern, but does not connect all the way through the gap
**2**	Moderate impression of a grating pattern, about half as strong as the reference grating
**3**	Strong impression of a grating pattern, though still weaker than the reference grating
**4**	Vivid impression of a grating pattern, as strong as the reference grating
**5**	Impression is stronger than the reference grating

## Data Availability

Data is available on https://osf.io/zfcw3/.
